# Arginine-Biofunctionalized Ternary Hydrogel Scaffolds of Carboxymethyl Cellulose–Chitosan–Polyvinyl Alcohol to Deliver Cell Therapy for Wound Healing

**DOI:** 10.3390/gels10110679

**Published:** 2024-10-23

**Authors:** Alexandra A. P. Mansur, Sandhra M. Carvalho, Ramayana M. de M. Brito, Nádia S. V. Capanema, Isabela de B. Duval, Marcelo E. Cardozo, José B. R. Rihs, Gabriela G. M. Lemos, Letícia C. D. Lima, Marina P. dos Reys, Ana P. H. Rodrigues, Luiz C. A. Oliveira, Marcos Augusto de Sá, Geovanni D. Cassali, Lilian L. Bueno, Ricardo T. Fujiwara, Zelia I. P. Lobato, Herman S. Mansur

**Affiliations:** 1 Center of Nanoscience, Nanotechnology, and Innovation—CeNano2I, Department of Metallurgical and Materials Engineering, Federal University of Minas Gerais, UFMG, Av. Presidente Antônio Carlos, 6627—Escola de Engenharia, Bloco 2—Sala 2233, Belo Horizonte 31270-901, MG, Brazil; alexandramansur.ufmg@gmail.com (A.A.P.M.); sandhra.carvalho@gmail.com (S.M.C.); nsvnadia@gmail.com (N.S.V.C.); gabrielagomes.ufmg@gmail.com (G.G.M.L.); 2 Laboratory of Immunobiology and Control of Parasites, Department of Parasitology, Institute of Biological Sciences, Federal University of Minas Gerais, UFMG, Belo Horizonte 31270-901, MG, Brazil; ramayana.brito@gmail.com (R.M.d.M.B.); isabelafbrito@gmail.com (I.d.B.D.); cardozzo.e.m@gmail.com (M.E.C.); bryanrihs13@outlook.com (J.B.R.R.); lilacerdabueno@gmail.com (L.L.B.); rtfujiwara@gmail.com (R.T.F.); 3 Department of Morphology, Institute of Biological Sciences, Federal University of Minas Gerais, UFMG, Belo Horizonte 31270-901, MG, Brazil; leticia.cristina93@gmail.com (L.C.D.L.); samarcos2005@yahoo.com.br (M.A.d.S.); 4 Laboratory of Compared Pathology, Department of Pathology, Institute of Biological Sciences, Federal University of Minas Gerais, UFMG, Belo Horizonte 31270-901, MG, Brazil; marinareys13@gmail.com (M.P.d.R.); cassalig@icb.ufmg.br (G.D.C.); 5 Chemistry Department, Federal University of Minas Gerais, UFMG, Belo Horizonte 31270-901, MG, Brazil; anapacheli@gmail.com (A.P.H.R.); luizoliveira@qui.ufmg.br (L.C.A.O.); 6 Departamento de Medicina Veterinária Preventiva, Federal University of Minas Gerais, UFMG, Belo Horizonte 31270-901, MG, Brazil; ziplobat@gmail.com

**Keywords:** skin substitutes, wound healing, biopolymers, living biomaterials, cell therapy

## Abstract

Wound healing is important for skin after deep injuries or burns, which can lead to hospitalization, long-term morbidity, and mortality. In this field, tissue-engineered skin substitutes have therapy potential to assist in the treatment of acute and chronic skin wounds, where many requirements are still unmet. Hence, in this study, a novel type of biocompatible ternary polymer hybrid hydrogel scaffold was designed and produced through an entirely eco-friendly aqueous process composed of carboxymethyl cellulose, chitosan, and polyvinyl alcohol and chemically cross-linked by citric acid, forming three-dimensional (3D) matrices, which were biofunctionalized with L-arginine (L-Arg) to enhance cellular adhesion. They were applied as bilayer skin biomimetic substitutes based on human-derived cell cultures of fibroblasts and keratinocytes were seeded and grown into their 3D porous structures, producing cell-based bio-responsive hybrid hydrogel scaffolds to assist the wound healing process. The results demonstrated that hydrophilic hybrid cross-linked networks were formed via esterification reactions with the 3D porous microarchitecture promoted by foam templating and freeze-drying. These hybrids presented chemical stability, physicochemical properties, high moisture adsorption capacity, surface properties, and a highly interconnected 3D porous structure well suited for use as a skin substitute in wound healing. Additionally, the surface biofunctionalization of these 3D hydrogel scaffolds with L-arginine through amide bonds had significantly enhanced cellular attachment and proliferation of fibroblast and keratinocyte cultures. Hence, the in vivo results using *Hairless* mouse models (an immunocompromised strain) confirmed that these responsive bio-hybrid hydrogel scaffolds possess hemocompatibility, bioadhesion, biocompatibility, adhesiveness, biodegradability, and non-inflammatory behavior and are capable of assisting the skin wound healing process.

## 1. Introduction

Skin, accounting for approximately 15–16% of body weight, is the largest organ in humans. It is responsible for protecting the body against external agents and is critical in regulating biological homeostasis. Among the multitude of skin functions (fluid loss and infection prevention, temperature regulation, tactile detection, etc.), wound healing is important for skin when injuries result in a loss of skin integrity and functionality [1,2,3].

Small wounds are repaired through the self-healing ability of the skin, which involves a cascade of integrated biological and molecular events of cellular response, extracellular matrix deposition, and remodeling, restoring the appearance and function of injured skin with minimal scarring. In case of full-thickness injuries and burns or under certain metabolic, pathophysiologic, and environmental conditions, this normal course of events does not occur, and the process of healing is impaired or delayed, leading to nonhealing wounds that are termed chronic wounds. Chronic wounds impact social interaction, mobility, and productivity. They also may lead to hospitalization, long-term morbidity, and even death, resulting in a loss of quality of life and a substantial economic burden on healthcare systems worldwide [1,2,3,4,5,6].

Conventional wound management includes surgical procedures and non-surgical therapies. Surgical treatment involves debridement as well as skin grafts and flaps. Skin grafts are used for large tissue damage and chronic wounds to cover the injured area with part of the dermis and epidermis harvested from healthy skin from the same person (autograft), from another person of the same species (allografts), or from a different species (xenografts). On the other hand, non-surgical therapies typically involve the use of pharmacological agents and dressings after proper preparation of the wound bed. These non-surgical therapies may address several functions, such as moisture regulation, exudate absorption, permeability to vapor/gas, autolytic or enzymatic debridement of the wound bed, control of re-epithelialization and angiogenesis, pain relief, and management of infections and inflammation, among others [2,3,6,7].

In the field of non-surgical treatment, products and strategies based on cell therapy have emerged in recent decades for large skin damages and chronic wounds to solve the problem of finite donor skin graft supply and the limitations involved in the use of skin, such as potential rejection, disease transmission, availability, restoration, scarring, and pain. The first studies on this involved the use of sheets of autologous keratinocyte cells, which evolved to the newer approach called “living biomaterials”, leading to the development of cellular tissue-engineered skin substitutes that combine cell transplantation with biomaterials. These smartly integrated biomaterials try to resemble skin layers and act as support for cell seeding but could also be biologically active and deliver growth factors [4,5,8,9,10]. Depending on the proportion of skin damage, different types of cellular tissue-engineered skin substitutes could be used: (a) cellular epidermal skin substitutes (usually keratinocytes cultured in biomaterial support); (b) cellular dermal skin substitutes (mainly fibroblasts delivered in a 3D porous matrix); and (c) epidermal–dermal bilayer cellular skin substitutes (epidermal and dermal layers with fibroblasts and keratinocytes). However, relatively few cellular products are already commercially available (e.g., Myskin^®^, Dermagraft^®^, Transcyte, Apligraf^®^, OrCel™, and Permaderm™), which are produced with both autologous and allogeneic human cells and approved for applications in some specific types of wounds. Nonetheless, several others are currently in distinct stages of development and clinical trials [2,3,5,7,11,12]. Among several challenges to be overcome in the multidisciplinary area of wound healing, one aspect of paramount importance is associated with the selection of the biomaterial to be used for manufacturing skin constructs. In the vast realm of the studies regarding wound dressings and skin substitutes reported in the literature [5,7,10,11,13,14,15], the first choices for materials have been collagen, hyaluronic acid, glycosaminoglycan, fibronectin, fibrin, elastin, amniotic membranes, and decellularized tissues and extracellular matrices (ECMs), focusing on resembling the native components of the skin.

Nevertheless, natural polymers and their derivatives (silk, gelatin, chitosan, agarose, carboxymethylcellulose, etc.) and synthetic polymers (polyurethane, silicon, polyvinyl alcohol, polyethylene glycol, polyhydroxybutyrate-co-hydroxyvalerate, etc.) have been increasingly researched [5,7,10,11,13,14,15]. Recently, a very comprehensive and systematic study developed by the U.S. Department of Health and Human Services in 2020 [12] identified 76 commercially available skin substitutes to treat chronic wounds in the United States, but only 8 (~11%) delivered cells. Regarding the biomaterials that behave as active functionals for cellular support and delivery, they were mostly based on human placental membranes (43%), animal tissue sources (28%), and donated (18%) or autologous (3%) human tissues. However, these types of biomaterials have raised concerns regarding their availability, potential diseases, and immuno-effects. Only two products (i.e., less than 3%) were of non-human or non-animal origin. In this view, there is an opportunity gap in the currently available wound therapies that should be addressed by developing new alternatives of cell delivery epidermal–dermal bilayer cellular skin substitutes without using human and animal tissue sources for biomaterial supports. Besides the aspects highlighted, these advanced smart biomaterials need to fulfill all challenging properties and features of skin tissue while also being from renewable and sustainable sources, at affordable costs, with worldwide availability to allow scalable and reproducible industrial production.

Thus, by envisioning the application of cellular skin substitutes, the constructs for cell seeding are expected to incorporate functions such as moisture/vapor regulation, exudate absorption, biocompatibility, porosity, cell attachment (bioadhesion), hemostasis, conditions for cell-to-cell communication, adhesiveness to the wound bed, mechanical properties, antimicrobial activity, and controlled biodegradation. Relying on these properties, the biomaterial should additionally provide a microenvironment and a 3D microarchitecture that mimics the native skin and favors skin repair with minimal scarring. In this field, porous hydrogels have garnered much attention due to a unique set of properties that could be tailored and improved by combining biopolymers with synthetic polymers. These hybrid systems may offer attractive enhanced functionality and properties that mimic natural tissues. Moreover, they could be further tuned by functional moieties for mediating and enhancing cell responses (e.g., peptides, growth factors, etc.) [2,3,6,7,11,15,16,17,18,19,20,21,22].

In this sense, polysaccharides and their derivatives have been considered the first biopolymer alternatives for tissue engineering applications, including chitosan (CHI) and carboxymethylcellulose (CMC) [5,7,15,18,19,23]. They are obtained from renewable sources, widely available, relatively low cost, and easy to handle and exhibit biocompatibility with and similarity to biological macromolecules and native tissue. Regarding synthetic polymers, polyvinyl alcohol (PVA) is a prevalent hydrogel preparation material in bioengineering. It is non-toxic, biocompatible, biodegradable, inexpensive, hydrophilic, and chemical-resistant [19,24]. Moreover, to achieve some specific biological requirements, rich amine-based biomolecules (i.e., -NH_2_ groups) have been reported to promote cell adhesion and proliferation [25,26]. In this field, it has been reported that L-arginine amino acid acts as a multifunctional biomolecule, favoring bioadhesion while also contributing in vivo to the secretion of nitric oxide (NO), which can support vascularization, enhancing insulin secretion in diabetic chronic wounds, combined with good anti-thrombogenic activity [27,28,29].

Hence, in this study, a novel type of biocompatible ternary hybrid hydrogel scaffold was designed, developed, and produced through an entirely eco-friendly aqueous process. The matrix of the hydrogel was composed of carboxymethyl cellulose, chitosan, and polyvinyl alcohol and chemically cross-linked by citric acid; this was followed by a freeze-drying process and biofunctionalization with L-arginine amino acid. In addition, a dermo-epidermal skin biomimetic substitute was obtained based on human-derived cell cultures of fibroblasts and keratinocytes seeded and grown onto these 3D matrices to produce cell-based responsive bio-hybrid hydrogel scaffolds to potentially assist in the wound healing process. These bio-hybrids were comprehensively investigated for their physicochemical, morphological, structural, and biological characteristics to act as 3D porous scaffolds for cell support and delivery assayed using in vivo mice models.

## 2. Results and Discussion

### 2.1. Design

**Figure 1:**
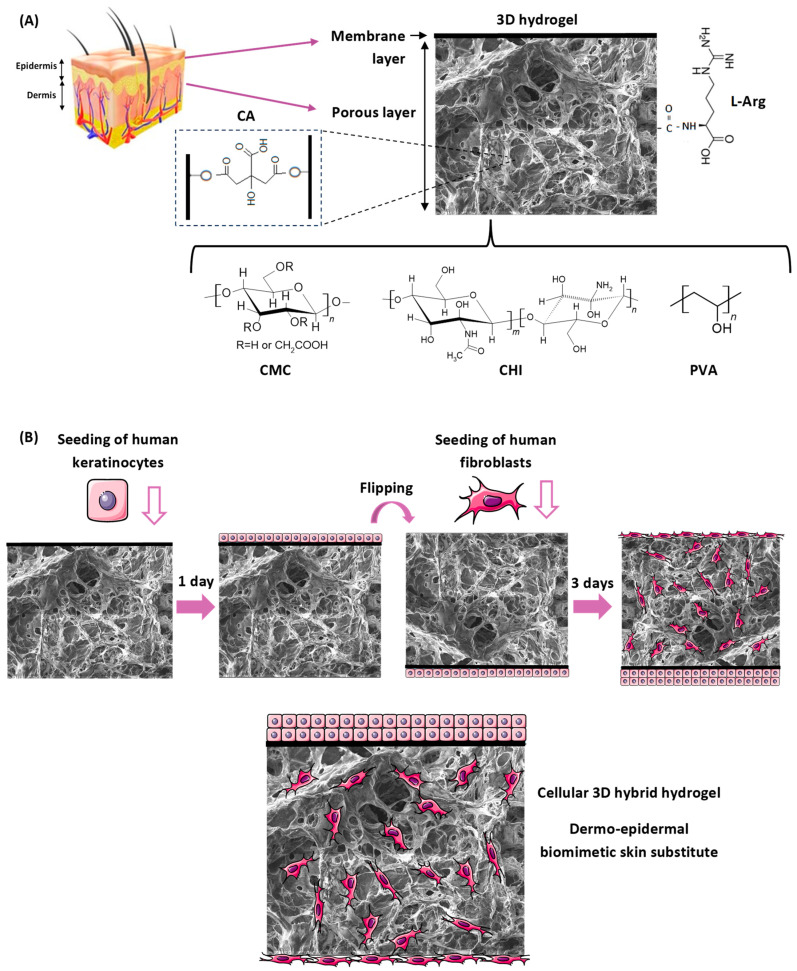
(**A**) An illustrative representation of a cross-section of the skin, indicating the layers (epidermis and dermis) that were resembled by the 3D hybrid hydrogel, depicted with information regarding its components, chemical formula, and structure. (**B**) Scheme of the co-culture strategy of the cell therapy product simulating the skin layers, with the deposition of keratinocytes in the membrane layer (resembling basement membrane of skin) and populating the porous layer with fibroblasts to obtain a cellular biomimetic dermal–epidermal skin substitute construct (not to scale).

A novel 3D construct was designed and developed through an advanced sustainable bioengineering strategy predominantly based on natural polymers (80% *w*/*w*), i.e., carboxymethyl cellulose (CMC) and chitosan (CHI), with stratified dermis and epidermis skin layers. It was biofunctionalized with L-arginine (L-Arg), incorporating autologous keratinocyte and fibroblast cultures for the repair/regeneration of the epidermis and dermis. A schematic representation is shown in Figure 1.

Carboxymethylcellulose is an anionic cellulose derivative where hydroxyl groups are substituted by carboxymethyl groups (-CH_2_-COOH), rendering water solubility for the biopolymer. CMC is also non-toxic, biocompatible, biodegradable, non-immunogenic, hydrophilic, tissue-resembling, available worldwide, and of low cost [18,19,23,30]. In order to control the physicochemical stability of the skin substitute, a CMC with a high molecular weight (700 kDa) and an intermediate degree of substitution (DS = 0.84) was selected for the production of the hydrogels as the main polymeric component (72% *w*/*w*).

Chitosan is a positively charged biopolymer obtained from chitin after the deacetylation process, producing a copolymer of N-acetylglucosamine and D-glucosamine units. Chitosan is non-toxic, biodegradable, biocompatible, mucoadhesive, and inexpensive. It also possesses bioadhesives, hemostatic and antibacterial/anti-yeast properties, and the soft texture of ECM that favors wound healing applications [15,31,32,33,34]. Chitosan (10% *w*/*w* of total polymer content) with a high molecular weight (Mw = 310–370 kDa) and an intermediate degree of deacetylation (DD = 75%) was the choice for this study in order to tailor skin substitute properties.

As reported in the introduction section, PVA is non-toxic, hydrophilic, biocompatible, and biodegradable. Also, it has mechanical properties that support cell adhesion, propagation, and migration, but PVA lacks sufficient bioactivity due to its synthetic nature. Therefore, PVA was selected to be combined with CMC and CHI biopolymers to form hybrid hydrogels with improved properties [19,35,36,37]. A PVA of medium molecular weight (85–124 kDa) and a high degree of hydrolysis (DH > 99%) was added at 20% *w*/*w* to enhance the physicochemical stability of hydrogels.

However, due to the high stability required for the application in the humid environment of the wound bed, it was not possible to comply only with electrostatic interaction and hydrogen bonds for physical cross-linking between polymers. The polymer chains were covalently bonded using eco-friendly, natural, and biocompatible citric acid (CA) as a cross-linking agent, avoiding the highly toxic crosslinkers that are often used, such as glutaraldehyde and epichlorohydrin. This chemical reaction was based on the formation of ester bonds between hydroxyl groups from the three polymers and citric acid at the mild thermal cycle used in this work (80 °C/24 h) [18,19,20].

The 3D template with interconnected porous and micrometric architectures resembling the ECM was obtained by combining the foam template method and a freeze-drying process. The foam template of hydrogels was provided by air entrapment in the liquid mixture during the mechanical mixing, which was stabilized in the aqueous medium by a PVA nonionic surfactant and solidified by the citric acid-mediated cross-linking reactions (the sample was identified as CMC:PVA:CHI_CA). The freeze-drying process was performed in the sequence (sample identified as CMC:PVA:CHI_FD).

In order to enhance the cell adhesion (i.e., bioadhesion) characteristics of the ternary hydrogel scaffold, which is vital for the wound healing process, binding sites based on amine (–NH_2_) groups were grafted onto the hydrogel surface [25,26,27,28,29]. L-arginine (L-Arg) amino acid was the functionalization molecule selected to favor cell attachment, and it was covalently conjugated to hydrogel carboxylic side groups using a 1-ethyl-3-[3-dimethylaminopropyl]carbodiimide (EDC) zero-crosslinking agent (sample identified as CMC:PVA:CHI_L-Arg).

Following this design, a bi-layered terpolymer hybrid hydrogel scaffold was successfully made in a one-pot synthesis to replicate the skin layers, with a surface membrane to mimic the basement membrane where keratinocytes were seeded and a porous layer to simulate the dermis for fibroblast population. The strategy for cell deposition for the co-culturing of the skin substitute is depicted in Figure 1B.

### 2.2. Polymer Characterization

**Figure 2:**
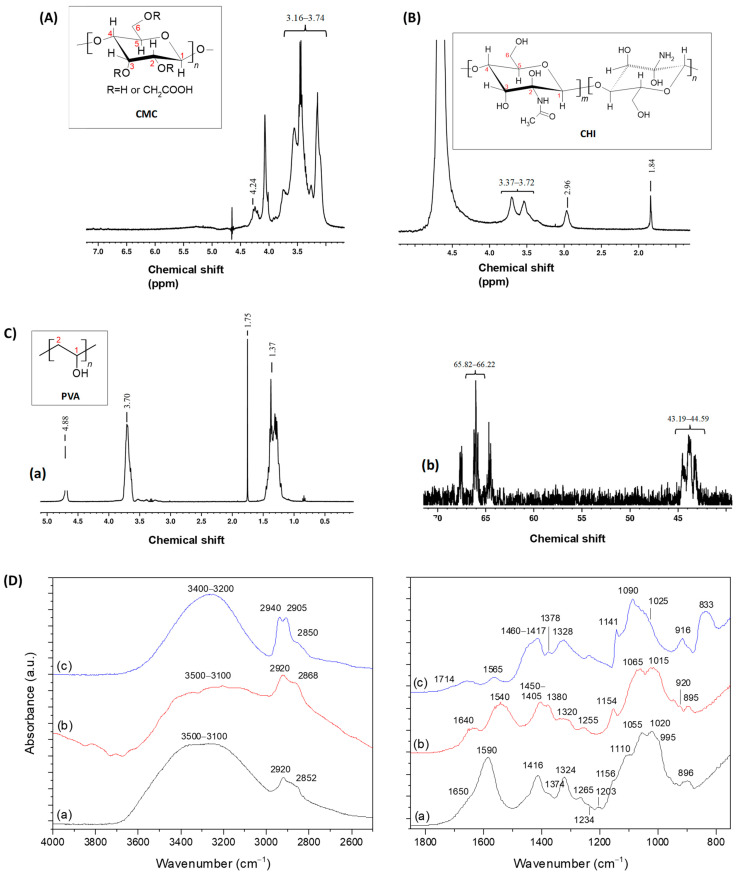
NMR spectra of (**A**) CMC (^1^H NMR), (**B**) CHI (^1^H NMR), and (**C**) PVA ((a) ^1^H NMR and (b) ^13^C NMR). (**D**) FTIR spectra of the three polymer components of hydrogels (a) CMC, (b) CHI, and (c) PVA.

Nuclear Magnetic Resonance (NMR) combined with Fourier-transform infrared spectroscopy (FTIR) techniques were employed to elucidate the chemical structure of the polymeric precursors. The sensitivity of NMR depends on factors such as the chosen nucleus, its abundance, and the chemical environment of the sample [38]. For CMC and CHI, ^1^H NMR was selected because of the high abundance of hydrogen nuclei, ensuring appropriate sensitivity for analysis. In the case of PVA, ^13^C NMR was also performed. Figure 2A–C shows the NMR spectra of CMC, CHI, and PVA, respectively.

In the ^1^H NMR spectrum of CMC (Figure 2A), the primary chemical shift peaks appeared between 3.16 and 3.74 ppm, corresponding to hydrogens bonded to C1, C3, C4, and C5 of the glycosidic ring. The signals between 4.08 and 4.64 ppm, particularly near 4.24 ppm, were associated with the protons of carboxymethyl groups (-O-CH_2_-COOD). Signals from 4.50 to 5.00 ppm were attributed to hydroxyl groups [39,40].

The CHI spectrum (Figure 2B) showed signals near 1.84 ppm corresponding to the three protons of N-acetylglucosamine, while the peak near 3.00 ppm was attributed to the C2-bonded proton of glucosamine rings. Chemical shifts from 3.37 to 3.72 ppm represented protons related to C3–C6, which, being non-anomeric and connected to the glycosyl ring skeleton, displayed comparable electron densities and similar chemical shifts. The H1 signals appeared at higher chemical shifts, ranging from 4.25 to 4.75 ppm, due to the electron-dense neighboring groups [34,40,41,42,43].

The ^1^H NMR spectrum of PVA (Figure 2C(a)) showed signals between 1.25 and 1.75 ppm, 3.50 and 3.75 ppm, and 4.75 and 4.90 ppm, corresponding to the protons of CH_2_, CH, and OH groups, respectively [38]. Moreover, the ^13^C NMR spectrum of PVA was collected to complement the analysis and is displayed in Figure 2C(b). The chemical shifts in the 65.82–66.22 ppm range were related to alcohol-bonded carbons present in C1 (CH-OH), while the peaks between 43.19 and 44.59 ppm corresponded to the secondary C2 signals (CH_2_) [44].

Notably, slight differences in chemical shifts for similar chemical groups were observable in the ^1^H NMR spectra of each analyzed sample. These differences arose because the spectrum of a polymer consists of a superposition of the spectra of its individual unit residues, which interact and form linkages with each other and with neighboring chains in specific ways, causing slight variations in the chemical shift peaks.

In the FTIR spectrum of CMC (Figure 2D(a)), the region between 3500 and 3100 cm^−1^ was credited to the stretching vibrations of O-H bonds. The νC-H region ranged from 3000 to 2800 cm^−1^. The contributions of ν_as_COO^−^ (1650 and 1590 cm^−1^) and ν_a_COO^−^ (1416 and 1324 cm^−1^) from carboxylate groups were observed as the pH of CMC solution is about 7.5, above de pKa of CMC. The region between 1400 and 1200 cm^−1^ also contained bands from δC-H (1374 and 1265 cm^−1^) and δO-H (1234 cm^−1^) vibrations, along with the symmetric (1203 cm^−1^) and asymmetric (1156 cm^−1^) stretching modes of the pyranose ring (C-O-C). The C-O vibrations from primary (C6-OH at 1028 and 995 cm^−1^) and secondary (C2-OH at 1110 cm^−1^ and C3-OH at 1055 cm^−1^) alcohols were also detected, with the band of glycoside bonds (β 1–4) observed at 895–900 cm^−1^. These findings highlight the key chemical groups of the CMC biopolymer, which feature typical saccharide rings linked by ether bonds, hydroxyls, and carboxymethyl groups grafted by the partial substitution of OH groups [18,19].

In the FTIR spectrum of the chitosan copolymer (Figure 2D(b)), peaks corresponding to νNH vibrations appeared in the 3500 to 3000 cm^−1^ region, overlapping with the stretch of hydroxyl groups. The bands at 2920 and 2868 cm^−1^ were ascribed to C-H stretching. The absorption at 1640 cm^−1^ was assigned to the carbonyl stretching of secondary amides (Amide I band) and the δN-H vibrations of the deacetylated primary amine (-NH_2_). The band at 1540 cm^−1^ was associated with the Amide II vibration. The bending vibrations of CH and OH appeared, ranging from 1450 to 1405 cm^−1^. The peaks at 1380 cm^−1^, 1320 cm^−1^, and 1255 cm^−1^ corresponded to the bending vibrations of the Amide III mainly associated with νCN and δNH. Additionally, bands at 1050–1100 cm^−1^ and 1040–1010 cm^−1^ indicated the C-O stretching vibrations in chitosan, corresponding to the C3-OH secondary alcohol and the C6-OH primary alcohol, respectively. The vibration at 894 cm^−1^ was assigned to the β-glycosidic bonds of chitosan [33,34,45].

The FTIR spectrum of PVA (Figure 2D(c)) contained contributions from vinyl alcohol (the main component, as the degree of hydrolysis was above 93%) and vinyl acetate units. The broadband between 3500 and 3200 cm^−1^ was associated with the νOH from the intra- and intermolecular hydrogen bonds between chains. IR absorption bands at 2940 cm^−1^ and 2905 cm^−1^ were related to νCH_2_ groups and the vibration at 2850 cm^−1^ was assigned to νCH_3_. Other significant bands of PVA included those associated with the vibrations of hydroxyls (C-OH) at 1328 cm^−1^ (δOH) and 1141 and 1090 cm^−1^ (νC-O), as well as alkyl groups (δCH_2_, 1460–1410 cm^−1^ and 916 cm^−1^) and ν(C-C) at 833 cm^−1^. The bands at 1714 cm^−1^ (νC=O), 1378 cm^−1^ (δCH_3_), and 1023 cm^−1^ (ν_s_ of =C-O-C) were from the remaining vinyl acetate units. The band at 1565 cm^−1^ was associated with remaining carboxylate groups (-COO^−^) from the saponification process [19,27].

### 2.3. Characterization of the Three-Dimensional Porous Hybrid Hydrogel Cellular Therapy Product

#### 2.3.1. Physicochemical and Morphological Properties

**Figure 3:**
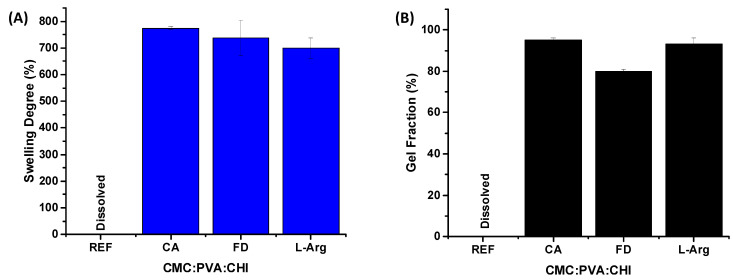
Results of (**A**) swelling degree (SD) and (**B**) gel fraction (GF) for 3D structures produced during different processing stages. Sample identification: REF refers to sample without citric acid (CMC:PVA:CHI); CA is the sample chemically cross-linked with citric acid (CMC:PVA:CHI_CA); FD is the cross-linked ternary hybrid hydrogel after the freeze-drying process (CMC:PVA:CHI_FD); and L-Arg is the cross-linked and freeze-dried sample after biofunctionalization with L-arginine (CMC:PVA:CHI_L-Arg).

The results for the swelling degree (SD) and gel fraction (GF) of the 3D constructs (scaffolds) during different stages of the preparation process are presented in Figure 3A and Figure 3B, respectively. Based on these findings, it was clearly observed that the 3D structures exhibited stability in an aqueous medium, which was attributed to the addition of citric acid as a cross-linking agent. In contrast, the sample without citric acid (CMC:PVA:CHI, reference REF) fully dissolved upon contact with water. The swelling degrees for the scaffolds across different production stages ranged from 700% to 800%. In comparison, gel fraction values exceeded 80%, reinforcing the role of chemical cross-linking in maintaining the equilibrium of the three-dimensional porous hydrogels. Therefore, the swelling degree and gel fraction results demonstrated that these 3D structures, prepared with CMC:PVA:CHI cross-linked with citric acid (CMC:PVA:CHI_CA), freeze-dried (CMC:PVA:CHI_FD), and modified with L-arginine (CMC:PVA:CHI_L-Arg), possessed suitable physicochemical properties necessary for biomedical applications in skin regeneration, where hydrophilic structures with high exudate retention and aqueous stability are crucial.

**Figure 4:**
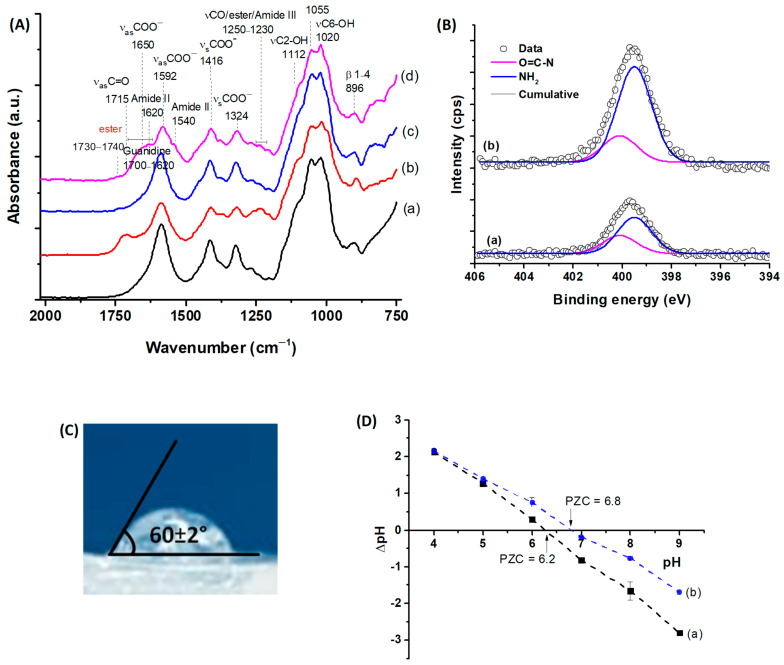
(**A**) FTIR spectra (range 2000–750 cm^−1^) for the samples: (a) CMC:PVA:CHI (REF), (b) CMC:PVA:CHI_CA, (c) CMC:PVA:CHI_FD, and (d) CMC:PVA:CHI_L-Arg. (**B**) XPS spectra of N 1s region for hydrogels: (a) CMC:PVA:CHI_FD and (b) CMC:PVA:CHI_L-Arg. (**C**) Surface wettability assessment of the 3D hydrogel CMC:PVA:CHI_L-Arg through contact angle measurement. (**D**) PZC studies for the samples: (a) CMC:PVA:CHI_FD and (b) CMC:PVA:CHI_L-Arg.

The evaluation of the chemical reactions at the various stages of production was conducted using infrared spectroscopy. The reference spectrum (CMC:PVA:CHI, Figure 4A(a)) primarily showed peaks corresponding to CMC, the main polymeric component of the system (CMC:PVA:CHI, 72:18:10 by mass), in agreement with the bands depicted in the previous Figure 2D(a). The presence of citric acid and the occurrence of cross-linking reactions (CMC:PVA:CHI_CA, Figure 4A(b)) were evidenced by the appearance of ester bond bands (1735 and 1250 cm^−1^) overlapped with the protonated CMC carboxylic acid bands (ν_as_C=O at 1715 cm^−1^ and νC-O at 1240 cm^−1^) [19].

After the freeze-drying stage (CMC:PVA:CHI_FD, Figure 4A(c)), due to the sample buffering process in PBS prior to the freeze-drying of samples, carboxylic groups were no longer observed (pH = 6.0–7.4 > pKa of CMC, R-COOH→R-COO^−^), highlighting the ester bond bands. In 3D samples functionalized with the amino acid L-Arginine (CMC:PVA:CHI_L-Arg, Figure 4A(d)), the Amide II peak was observed at 1540 cm^−1^ (δNH and νCN), associated with the formation of amide bonds (N-C=O) mediated by EDC. The Amide I band (1640 cm^−1^, νC=O) and Amide III band (1240 cm^−1^, νCN and δNH) overlapped the broadband characteristic of L-Arg guanidine groups (1700–1620 cm^−1^) and the ester bonds from chemical cross-linking with CA, respectively [27].

To further demonstrate the effective surface biofunctionalization of the 3D constructs by L-arginine (L-arg), X-ray photoelectron (XPS) spectra of the N 1s region of hybrid hydrogels were obtained before (CMC:PVA:CHI_FD) and after (CMC:PVA:CHI_L-Arg) grafting of the amino acid (Figure 4B). The deconvoluted high-resolution spectra for unmodified scaffolds showed two major peaks at binding energies of 400.1 eV and 399.5 eV. Both bands were associated with chitosan biopolymer. The peak at 400.1 ± 0.2 eV was attributed to unprotonated amine (-NH_2_) and the component at 399.5 ± 0.2 eV to the O=C-N group, present due to the incomplete deacetylation of chitosan (DD = 75%) [46,47]. After L-arginine surface grafting, an increase in the intensity of both bands was detected due to the incorporation of new -NH_2_ groups from L-Arg and the amidation reaction of crosslinking based on EDC. It was estimated that there was an increase in concentration in the mass (wt%) of nitrogen atoms on the surface from 2% to 5% (Vision 2.0 Processing software, Kratos, Kawasaki, Japan).

The hydrophilic/hydrophobic behavior of the amino acid-functionalized 3D hydrogel was evaluated using goniometry through static contact angle measurements (sessile drop, Figure 4C). The results indicated a contact angle of 60°, which aligns with the optimal range reported for promoting cell attachment and proliferation (55–85°) [48].

To further support biofunctionalization analysis, point of zero charge (PZC) measurements of the hydrogels were performed to evaluate the surface charge of the constructs (Figure 4D). The PZC values were approximately 6.2 and 6.8 for CMC:PVA:CHI_FD and CMC:PVA:CHI_L-Arg, respectively, as indicated by the points of intersection at the line of ΔpH = 0. This shift of PZC to higher values reflected the effect of grafting L-arginine, increasing the amine-based positively charged functional groups on the hydrogel surface. At neutral conditions, the carboxylic acid of L-arginine is deprotonated (R-COO^−^, pKa < 2.5), the amino group is protonated (NH_3_^+^, pKa~9), and the side group is in the guanidinium form (-C-(NH_2_)_2_^+^, pKa > 12). After the reaction of the amine of L-Arg with carboxylate groups of CMC forming amide bonds, the amino acid still contributed to amine positively charged moieties, increasing PZC values [49,50].

In addition to surface and physicochemical properties, morphological aspects such as the porosity and pore size of the 3D structures intended for tissue engineering applications were crucial for the biological properties of the developed constructs.

**Figure 5:**
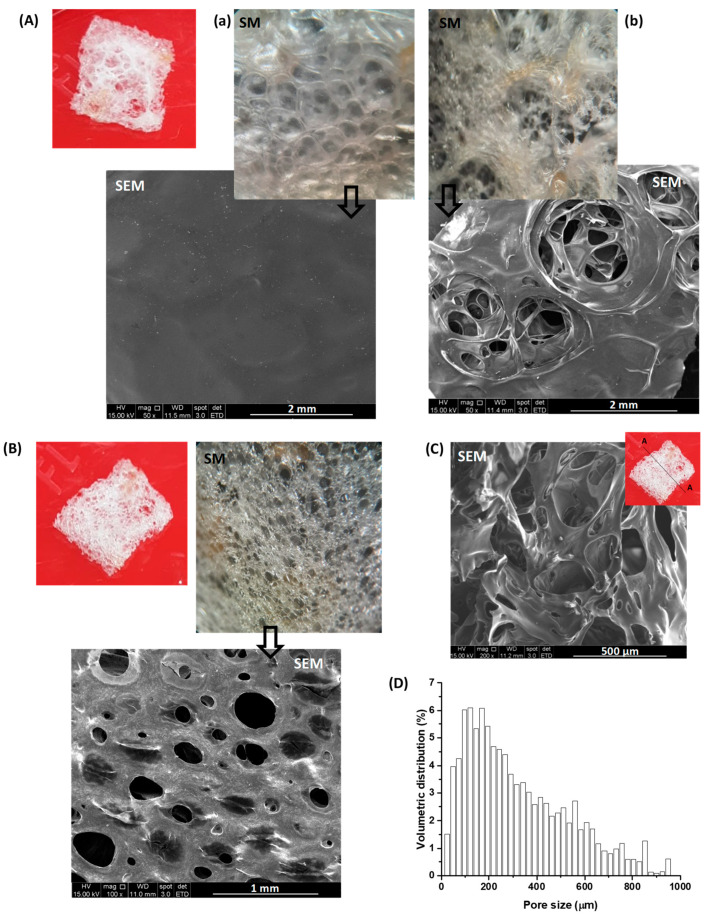
Digital images (red background), stereoscopic microscope images (SM, 20×), and scanning electron microscopy images (SEM, 50×–100×) of the (**A**) upper and (**B**) lower surfaces of the porous three-dimensional structure. (**C**) SEM image (200×) of cross-section (Section A-A in the detail of the digital image). (**D**) Volumetric pore size distribution by micro-CT.

In this project, images from stereoscopic microscopy (SM), scanning electron microscopy (SEM), and X-ray microtomography (micro-CT) analyses were used to evaluate the thickness, morphology, and porosity of the developed 3D structures. Based on these analyses, confirmed by caliper measurements, the structures exhibited a thickness of 1 to 2 mm. Stereoscopic images, in combination with SEM, indicated that the upper surface of a 3D structure (the top side during the drying/cross-linking/lyophilization processes) had a closed surface (like a membrane, Figure 5A(a)), with very few openings, which were approximately 1 to 2 mm in size (Figure 5A(b)). Through these openings, a highly interconnected porous structure with pore sizes typically ranging from 150 to 500 μm was visible. On the scaffold’s bottom surface (in contact with the Petri dish during the drying/cross-linking/lyophilization processes), pores ranging from 50 to 600 μm were observed (Figure 5B), which is appropriate to allow fibroblast infiltration upon seeding (fibroblast length 150 to 200 µm and average width 15 µm) [51]. The cross-sectional image (Figure 5C) confirmed the formation of a three-dimensional sponge-like pore structure with observed multi-dimension interconnected porosity.

X-ray microtomography analysis revealed porosity ranging from 75% to 82%, nearly 100% relative to open pores, and pore size distribution, indicating that about 60% of the pore volume was in the range of 150–600 μm (Figure 5D). Open porosity and pore interconnectivity are essential for new tissue formation, cell infiltration and proliferation, nutrient and waste exchange, cell-to-cell communication, and vascularization. Generally, high porosity and pore sizes greater than 100–250 μm promote cell proliferation, extracellular matrix component secretion, and angiogenesis [11,17,52].

Thus, the morphology/porosity results demonstrated that the developed three-dimensional constructs (3D scaffolds) exhibited, in addition to physicochemical stability, a structure with hierarchical pore distribution and dimensions, connectivity, and surface area suitable for supporting cellular processes such as bioadhesion, cell proliferation, and biological medium flow (bioreactor), among others, which are crucial for epithelial tissue engineering applications. This set of properties and characteristics confirmed that the design, synthesis, characterization, and production process of the 3D scaffolds could aid and promote the formation, repair, and growth of new tissues in skin lesions and wounds.

#### 2.3.2. Biological Properties and Characteristics

**Figure 6:**
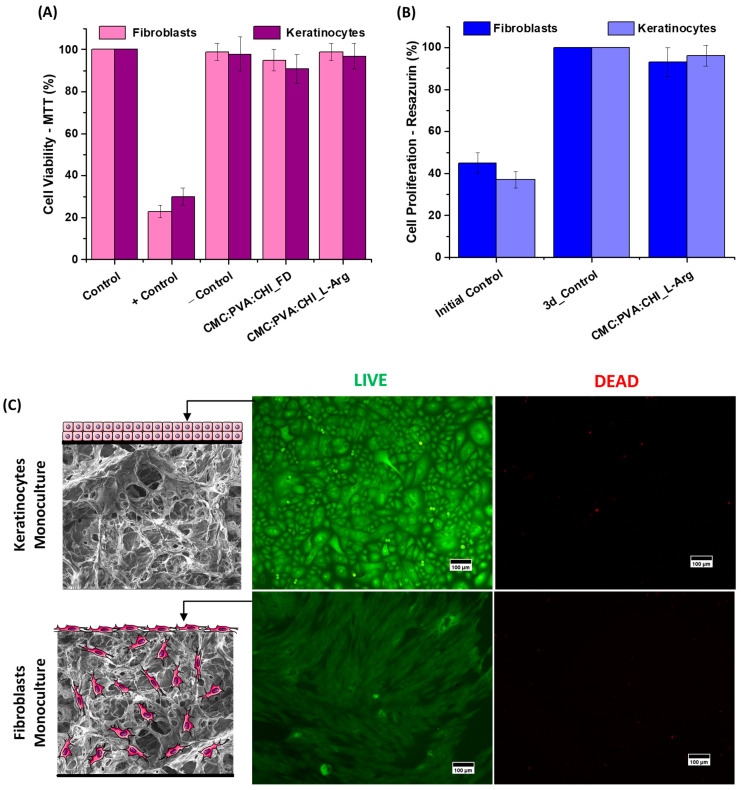
(**A**) Results of cell viability (MTT cytotoxicity test) and (**B**) cell proliferation (measured using resazurin protocol) biological assays for the porous three-dimensional structures (3d = 3 days). (**C**) Calcein AM (viable cells, green fluorescence, left side images, identified as LIVE) and ethidium homodimer (dead cells, red fluorescence, right side images, identified as DEAD) staining for keratinocyte and fibroblast monoculture cells deposited onto the lower and upper faces of the L-arginine functionalized 3D constructs, respectively (scale bar = 100 µm). Details: schematic representation of cell deposition (not to scale).

Cell viability assays on the scaffolds were performed using dermal cells (human primary keratinocytes) and epidermal cells (human primary fibroblasts). Results for the CMC:PVA:CHI scaffold samples before (CMC:PVA:CHI_FD) and after (CMC:PVA:CHI_L-Arg) functionalization with amino acid, evaluated by MTT (3-(4,5-dimethyl-2-thiazolyl)-2,5-diphenyl-2H-tetrazolium bromide) assay, showed that the developed structures were non-cytotoxic according to the international standard ISO 10993-5:2009 [53], with cell viability >90%, after 24 h of contact with the samples (Figure 6A).

Hemocompatibility is another key property when considering biomaterial suitability for skin tissue engineering constructs. Basically, materials are classified as non-hemolytic when the hemolytic index (HI) is below 2, slightly hemolytic when the HI ranges from 2 to 5, and hemolytic when the HI is higher than 5, according to ASTM F756-00 [54]. The hemocompatibility results of the scaffolds (CMC:PVA:CHI_FD and CMC:PVA:CHI_L-Arg) showed HI values below 1%, thus indicating that the 3D scaffolds were non-hemolytic.

For the bioadhesion and proliferation evaluations, seeding of each cell type was performed in the 3D structures separately (cellular monoculture). Considering the proposed model for the bi-layer skin tissue engineered construct depicted in Figure 1, keratinocyte cells were deposited on the upper face of the scaffold. This selection was based on the morphology and structure of the scaffold, with a relatively continuous surface with lower porosity acting as the basement membrane for the development of the stratified epidermal layer of keratinocytes but allowing the diffusion/exchange of nutrients and biological cues (cell-to-cell communication, cytokines, growth factors, etc.), which are crucial for the repair of the skin layers [10,15,55]. Fibroblasts were deposited on the lower face due to their porous morphology to act as a hosting structure for cellular invasion/infiltration, ECM deposition, and capillary growth (i.e., vascularization) [1,2].

A resazurin assay was performed to quantitatively evaluate the cell proliferation capacity of dermal and epidermal cells in the functionalized 3D structures (CMC:PVA:CHI_L-Arg) (Figure 6B). This assay is usually applied to measure the number of live cells in a sample [56]. After 3 days of fibroblast or keratinocyte seeding in the 3D structures, the results indicated that cell proliferation occurred in the materials, with an increase in the number of cells on the third day (93% for fibroblasts and 96% for keratinocytes), similar to that observed for the control after 3 days (3-day control, in the graph, set as 100% for both cells). Compared to the initial day (initial control in the graph, 45% for fibroblasts and 47% for keratinocytes), the number of cells doubled. It should be observed that for the 3D construct sample without L-arginine functionalization (CMC:PVA:CHI_FD), after 3 days of contact with fibroblasts, cell proliferation measured by resazurin was only 16%—lower than the initially seeded value (~45%). These results are crucial for the development of cellular therapy systems, where cells are seeded on three-dimensional scaffolds to produce so-called cellular bio-hybrids (“living biomaterials”). Also, these outcomes demonstrated that L-arginine biofunctionalization was crucial for enhancing cell attachment to the biomaterial matrices, leading to proliferation.

The results of cell adhesion in the scaffolds (bioadhesion) and the cytocompatibility of the 3D structures functionalized with the L-arginine (CMC:PVA:CHI_L-Arg) could be visualized in the fluorescence microscopy images of each face of the construct after 3 days of monoculture seeding using the LIVE/DEAD kit. In Figure 6C, the live cells fluoresce in green (i.e., emission from calcein AM cell-permeant dye after hydrolysis by intracellular esterases) and the dead cells in red (i.e., emission of ethidium homodimer-1 cell-impermeant viability indicator that selectively stains cells with damaged plasma membranes upon binding to DNA) [32]. For both lines, it could be seen that adherent live keratinocytes and fibroblasts predominated and were well distributed in the upper and lower faces of the hydrogel, respectively. The high content of live cells stained with calcein AM and the absence of cells stained in red also corroborated that the 3D-functionalized hydrogels were cytocompatible and rendered an appropriate hosting structure for cell seeding. As a reference, for the 3D constructs without L-arginine biofunctionalization (CMC:PVA:CHI_FD), no well-distributed layer of live fibroblasts and keratinocytes could be observed, indicating poor cell attachment and adhesion to these unmodified matrices. These findings validated the previous results regarding the efficiency and vital importance of L-arginine amino acid grafting in the surface of hybrid terpolymer structures to the development of this cellular skin substitute.

Therefore, the results of cell viability assays (MTT), hemocompatibility, resazurin assessment, and fluorescence microscopy confirmed that the three-dimensional scaffolds based on CMC:PVA:CHI_L-Arg were non-cytotoxic and biocompatible and that bioadhesion and proliferation were achieved.

#### 2.3.3. Development of 3D Cell Therapy Product as a Dermal–Epidermal Skin Substitute

The acellular and cellular in vitro results described in the previous sections indicated that the developed hydrogel biomaterials presented physicochemical (swelling capability, gel fraction, hydrophilicity, and surface charge and chemistry), morphological (high porous interconnected microarchitecture), and biological (non-cytotoxic, non-hemolytic, cell attachment, and proliferation) properties necessary for producing an appropriate cellular therapy system to be used in the treatment of skin wounds. Based on these promising results, the functionalized porous three-dimensional structure CMC:PVA:CHI_L-Arg was used as a polymeric substrate for the development of the bilayer cell therapy product for seeding the co-culture of keratinocytes and fibroblasts for the replacement of lesions in the dermis and epidermis.

In the process of cell seeding, the polymeric skin construct became swollen in contact with the biological medium, resulting in a soft and flexible material. Additionally, during all the steps involving the cell culture and after the end of the process, the structures remained intact and easy to handle.

**Figure 7:**
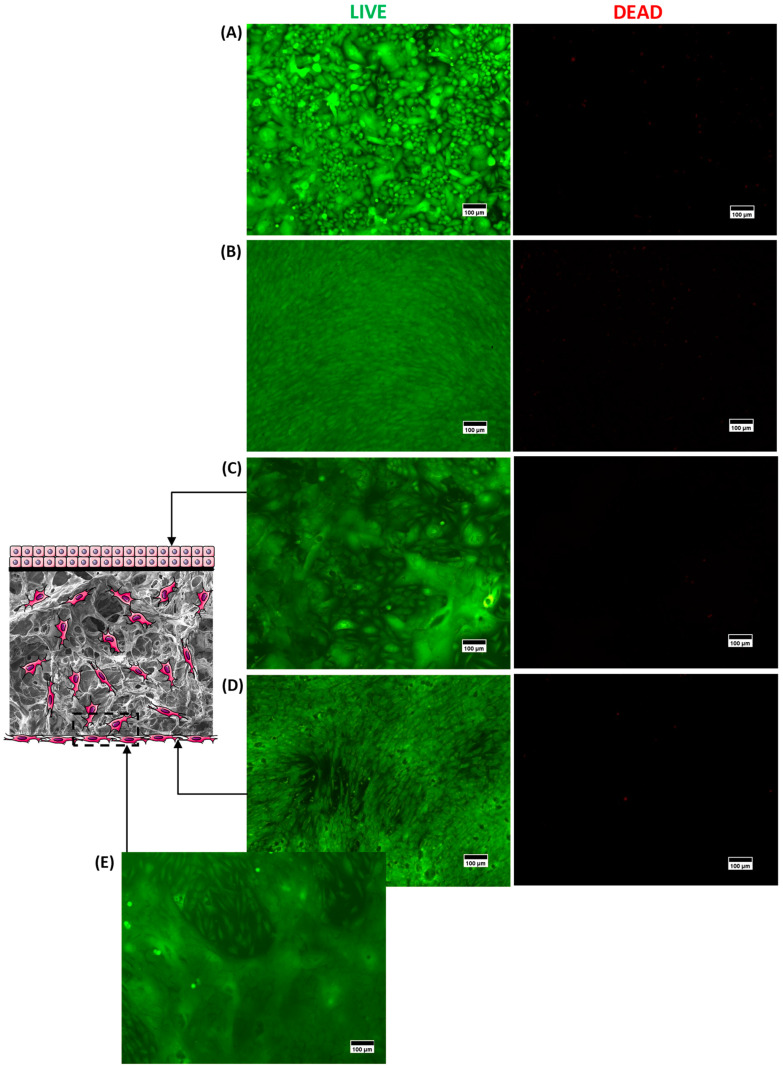
Fluorescence microscopy images showing representative images of the 3D CMC:PVA:CHI_L-Arg structure, where a co-culture of (**C**) keratinocytes (upper face—membrane) and (**D**,**E**) fibroblasts (lower face—porous structure) were seeded compared to controls ((**A**) Keratinocyte +Control and (**B**) Fibroblast +Control). Cells were labeled with calcein AM (viable cells, green fluorescence, left column, identified as LIVE) and ethidium homodimer (dead cells, red fluorescence, right column, identified as DEAD). Scale bar = 100 µm. Detail: schematic representation of cell therapy dermal–epidermal skin substitute (not to scale).

The fluorescence microscopy results of the LIVE/DEAD intracellular viability marker for the upper (Figure 7C, keratinocytes) and lower (Figure 7D, fibroblasts) faces of the same 3D structure are presented in Figure 7. For each side of the construct, live cells (green fluorescence, LIVE column) were obtained and uniformly distributed in the material with a near absence of dead cells (red fluorescence, DEAD column), indicating the presence of adherent live cells in both faces of the scaffold. Moreover, the morphology of each culture was comparable to its respective positive control (cells cultivated in the well, without 3D structures; polystyrene, flat base, standard for adherent cells) for keratinocytes (Figure 7A) and fibroblasts (Figure 7B). In addition, for fibroblasts (Figure 7D,E), the invasion of cells into the scaffold porosity is clearly depicted in Figure 7E, where the green emission of live fibroblasts could be observed. In this sense, these results showed that the seeding process of both cells in 3D scaffolds was effective, as proposed in the scheme for co-culture depicted in Figure 1B.

These results are very important as they demonstrated that a bilayer tissue-engineered skin bio-hybrid was obtained with affordable and highly available polymers and reagents based on relatively simple synthesis steps and biofunctionalized with a single amino acid. As no human or animal tissue sources were used, these biomaterial substrates based on two modified polysaccharides do not raise concerns regarding immune effects and the transmission of diseases. Additionally, the presence of adherent layers of keratinocytes and fibroblasts could assist wound healing. Keratinocytes mainly secrete growth factors and cytokines that promote cell proliferation and migration, respectively. Fibroblasts, besides delivering various cytokines and growth factors that participate in cell proliferation processes, the development of blood vessels, and inflammatory processes, also produce three-dimensional ECM (proteoglycans, collagens, and other proteins). The presence of both dermal (fibroblasts) and epidermal (keratinocytes) transplanted cells in the product, due to cell-to-cell communication, also favors the repair of the skin layers by promoting epithelial stratification, increased tensile strength, the modulation of cytokines, and growth factor expressions, with enhanced angiogenic properties [6,55,57].

During this preclinical development, allogeneic human foreskin fibroblasts (h-Fb) and keratinocytes (h-KT) from a cell bank were used as an allogeneic model. This kind of cell has been widely utilized in various commercial cellular skin substitute products (e.g., Apligraf^®^, OrCel™, Dermagraft^®^) approved by the U.S. Food and Drug Administration Authority (F.D.A.) for application in wound healing treatments. However, when cell therapy products are developed and used, there is always a concern about the source of the cell lines (allogeneic x autologous) due to the pros and cons of each type. Autologous cells do not require human leukocyte antigen (HLA) matching or immune-suppressive drug therapy and have a low risk of other diseases, but harvesting enough cells needed for the therapy is very expensive and time-consuming (for example, approximately 2–4 weeks of culture from biopsy for sufficiently sized graft). On the contrary, allogeneic cells are readily available from cell banks. They are scalable from a manufacturing perspective to produce high-quality, affordable products in accordance with good manufacturing practice (GMP) regulations. Its use is also favored by youth and healthy donors, eliminating the co-morbidities associated with the recipient. The drawbacks include the restriction of donors, reduced cell viability after infusion, and potential immune rejection [5,16,58]. More specifically, regarding skin damage applications, the use of autologous cells is associated with an acceleration of wound healing due to the lower time required for the host cells to invade the wound bed and the early synthesis of new skin. Conversely, allogeneic cells do not attach and cover the wound permanently, and graft survival is usually short-term (4–8 weeks) due to the death of cells. Nevertheless, they release growth factors, ECM, and basement membrane components that promote host cell migration and proliferation from the wound edges (accelerating epithelialization) and beds (promoting granulation formation) [5,8,10].

Although the major goal was designing and developing, as a proof of concept, a novel skin substitute for cell therapy, one should bear in mind its potential future clinical applications. Thus, the biomaterial hybrids developed in this work, which were designed to be immune-compatible, could be prepared and tested using sterile reagents and under aseptic conditions. Ideally, it could be used to transplant autologous or allogeneic cells to the wound bed. Autologous cells or HLA-matched donor cells could be prepared from biopsies and seeded in this skin substitute following the same strategy. If necessary, as there are some restrictions for the use of human samples from biobanks for commercial purposes [59], allogeneic cells could be isolated, extensively tested, and stored for future use, for example, those from discarded skin tissue obtained from healthy patients undergoing abdominoplasty [10]. As the biomaterials are readily available, the biocomposite cell structure could be seeded with the desired cells in only 4 days (after cell culture availability), providing a fresh product for use as a replicate of full-thickness skin grafts, including a layer of keratinocytes resembling the epidermis and a porous structure populated with fibroblasts as the dermis. In this sense, the selection of the type of cell to be used will be more dependent on the possibility of self-donation and the urgency of the skin tissue replacement. It is important to highlight that although these results are very promising, further studies are required to properly overcome the many challenges in this area of research, such as the high complexity of the skin wound healing process in healthy and ill patients, shelf life and long-term stability, and the strict approval processes of regulatory agencies, before allowing these innovative bio-hybrid scaffolds to be approved for clinical trials in humans and animals.

### 2.4. Evaluation of Cell Therapy Products in Immuno-Compromised Hairless Mice

In vivo tests were performed to move to the next step in the development of the biomedical skin substitutes. These bioassays were vital to analyze the capacity of the cell membranes (CM, dermo-epidermal skin tissue engineering constructs with keratinocyte and fibroblast co-culture) to stimulate tissue healing in an experimental model of immune deficiency, *Hairless* (hr/js) mice. These animals had an impaired T lymphocyte response due to the total absence (or atrophy) of the thymus in response to the genetic mutation on chromosome 14. These *Hairless* mice were given well-defined lesions on their backs and were monitored for 14 days. Cellular skin substitute hydrogels were tested in comparison to the control group (without hydrogel, identified as control), where the lesion was covered with a “Blood Stop” dressing.

Several methods have been used for the assessment of the evolution of the wound healing process, according to the literature [60,61]. For example, the scoring method has been an option to qualitatively evaluate the wound healing process. On the other hand, the relative wound area (e.g., calculated from the diameter of a lesion) and wound healing rate (WHR, %) have been more broadly applied to evaluate the evolution of the cicatrization process in in vivo animal models.

**Figure 8:**
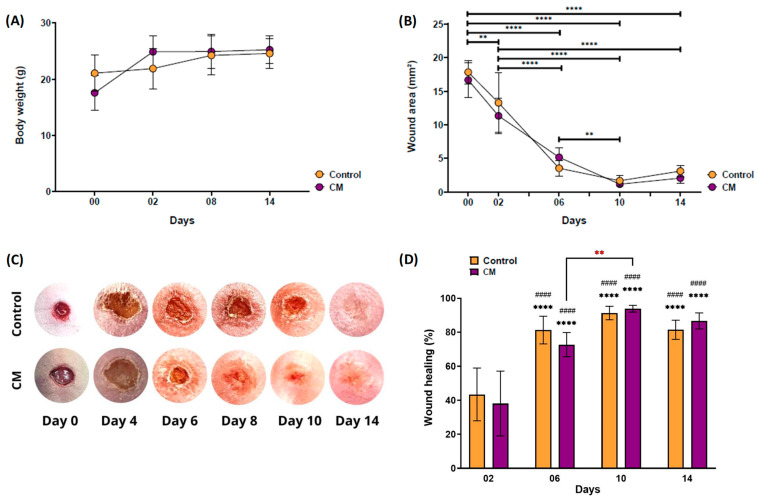
(**A**) Monitoring of the body weight of the mice after inducing skin lesions and applying a cell membrane (CM group) compared to the control group (without membrane). (**B**) Measurement of the wound area over 14 days for both the cell membrane and control groups. (**C**) Representative photos showing the wound healing process during the days of the experiment and (**D**) percentage of wound healing (WHR, %). The control group consisted of healthy female *Hairless* mice (n = 8), with their wound covered by a “Blood stop” bandaid but with no membrane applied. The CM group included *Hairless* mice with wounds covered by the tested cell membrane (CM, n = 8). In the graph plot D, (*) indicates statistical differences where all groups on days D06, D10, and D14 were compared to the control group on day D02. (#) Indicates statistical difference where all groups on days D06, D10, and D14 were compared to the membrane-treated (CM) group on day D02. The red asterisk represents the statistical difference between the membrane-treated groups (CM) at D06 and D10. Data are shown as mean ± standard deviation. **/## *p* < 0.01; ****/#### *p* < 0.0001.

Clinically, the mice did not show significant changes in their body weight after wound induction (Figure 8A). Additionally, the animals did not show signs of rejection of the membranes nor visible signs of an inflammatory process afterward. All membranes remained fixed until the end of the 14 days of monitoring, in agreement with the requirements of proper adhesiveness to the wound bed recommended for skin substitutes [62]. More importantly, it was possible to qualitatively and quantitatively observe the reduction in the lesion area (Figure 8B,C) over time.

Despite the fact that the percentage of wound healing between the groups analyzed was significantly higher from D06 onward than from D02 (Figure 8D), there was no statistical difference between the control group without membrane and the group treated with CM at each time analyzed. The animals that received the cell membrane presented a significantly higher healing rate at D10 compared to the same group at D06. It was possible to observe that the percentage of healed area remained above 80% and 70% for both the control group and the CM membrane group, respectively, from D06 onward. Thus, it should be highlighted that from these in vivo results, comprehensively corroborated by the previous biological in vitro assays, the novel 3D scaffolds were capable of contributing to healing without leading to tissue impairment observations or causing visible rejection events when applied to the wound injuries in mice with deficient cellular immune responses.

**Figure 9:**
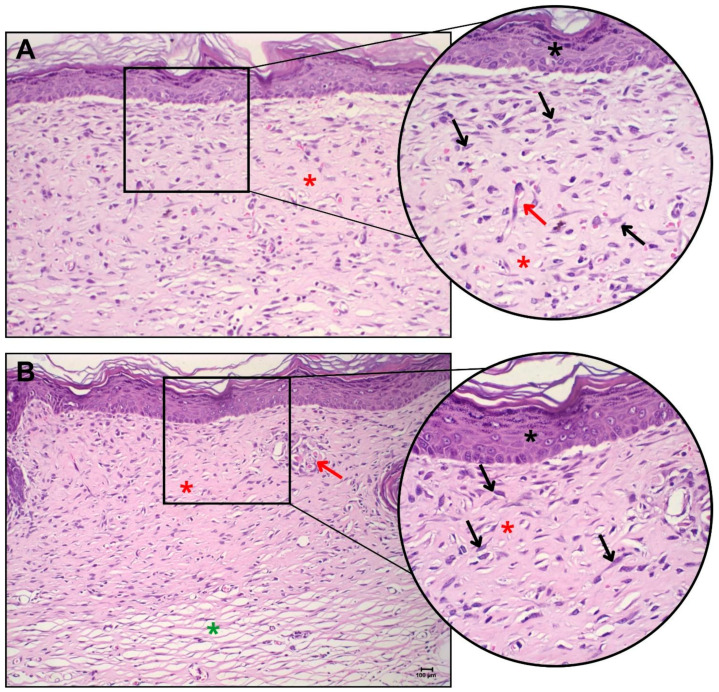
Histopathological analysis of skin lesions in *Hairless* mice, showing representative images of (**A**) skin from the control group (no hydrogel applied) and (**B**) skin from the group treated with the 3D bilayer construct containing fibroblasts and keratinocytes (CM group). Black asterisk: intact epidermis; red asterisk: denser collagen; green asterisk: looser collagen; black arrow: reactive fibroblasts; red arrow: blood capillaries. The larger image displays the lesion area at 20× magnification, with a zoomed-in view of the highlighted area at 40× magnification.

In the histopathological evaluation of the *Hairless* animals, complete healing of the injured area could be observed for both groups (Figure 9). Among the animals in the control group (Figure 9A), without the application of the cellular skin bilayer construct, it was possible to observe the area of injury with complete healing, with total recovery of the epidermis and dermis, absence of ulcerations, and no inflammatory infiltrate. At 40× magnification, there was discrete late granulation tissue, with the presence of dense collagen, in the remodeling process.

There was the presence of cells compatible with reactive fibroblasts with stellate/fusiform morphology. In addition, some blood capillaries perpendicular to the epidermis were observed, which characterized the granulation tissue.

In the group treated with the cell membrane (dermo-epidermal skin tissue engineering constructs with keratinocyte and fibroblast co-culture, Figure 9B), it was possible to visualize the intact and fully recovered epidermis and dermis. Rare inflammatory foci were observed, with the presence of denser collagen and areas of looser collagen in the innermost portion of the tissue. There was the presence of reactive fibroblasts and the absence of granulation tissue, indicating complete recovery of the tissue.

Hence, these results indicated that the innovative bio-hybrid hydrogels designed and produced in this work matched the requirements of biocompatibility and biodegradation properties and did not impair neovascularization or skin repair.

Finally, it is important to point out that, to the best of our knowledge, this is the first report to design, develop, and produce 3D porous ternary polymeric matrices predominantly composed of sustainable and renewable resources (i.e., two biopolymers, CMC and CHI, 80%), through an aqueous process, under mild conditions (e.g., T, pH), with no harmful precursor reagents. Moreover, instead of using common toxic chemical crosslinkers (such as glutaraldehyde, epichlorohydrin, etc.), these hydrogel networks were created with citric acid as the crosslinker, a water-soluble, biocompatible, and renewable resource. Furthermore, the surfaces of these 3D hydrogel scaffolds were functionalized by a biocompatible and environmentally friendly biomolecule, L-arginine. In this view, bearing in mind the concept of developing novel biomaterials for assisting wound healing and skin tissue engineering, the authors moved beyond the scientific and technical requirements by also adopting a biosafe and sustainable strategy in the entire development process.

## 3. Conclusions

In summary, dermo-epidermal tissue engineered 3D porous scaffolds were successfully designed and produced based on biocompatible ternary hybrid hydrogels. They were made of carboxymethyl cellulose, chitosan, and polyvinyl alcohol; chemically crosslinked by citric acid based on an environmentally benign aqueous route; and subjected to foam templating and freeze-drying porogenic processes. These 3D scaffolds were effectively surface biofunctionalized with L-arginine by an amidation reaction. They showed a set of characteristics appropriate for wound repair applications, including swelling degree (~700%), gel fraction (>90%), and physicochemical stability. Moreover, they displayed morphological features comprising a continuous surface and a porous layer (75–80% open porosity, predominantly ranging from 150 to 600 μm), which permitted cellular infiltration, vascularization, and nutrient exchange, which are vital in assisting wound healing. These 3D scaffolds were non-cytotoxic and non-hemolytic and, favored by L-Arg surface functionalization, enhanced bioadhesion and the proliferation of keratinocytes and fibroblasts. They behaved in vitro as “living biomaterials” by seeding human-derived keratinocytes and fibroblasts in their three-dimensional porous structure, building an active dermo-epidermal bilayer. The in vivo results of the cellular therapy for assisting wound healing applications using a *Hairless* mice model demonstrated that the wound healing process was effectively achieved after applying the cellular membrane. No induced inflammatory processes or detectable biological rejections were observed, confirming their biocompatibility. More importantly, all mice specimens with open full-thickness wounds were able to progress in the healing process, achieving complete wound closure by day 14 post-lesion induction, with a fully recovered epidermis and dermis, validated by histopathological analysis. Hence, based on the positive results achieved in this study, it can be envisioned that the developed cellular dermo-epidermal skin tissue engineered constructs, as a novel bio-hybrid scaffold, have the potential to be applied as a viable skin substitute system to assist the wound healing process. Nonetheless, further studies are needed to overcome the numerous challenges in this field, including the complexity of the skin wound healing process, shelf life and stability, and the strict approval processes of regulatory agencies, before allowing these promising bio-hybrid scaffolds to be approved for clinical trials and biomedical applications in animals and humans.

## 4. Materials and Methods

### 4.1. Materials

All precursors and reagents are described, including their sources/manufacturers, the first time they appear in the next sections. They were used as received, without further purification. Unless specified otherwise, deionized water (DI water, resistivity of 18 MΩ∙cm, Millipore Simplicity^TM^, Merck Millipore, Burlington, MA, USA) was used to prepare the aqueous solutions. Unless specifically mentioned, all procedures were performed at room temperature (RT, 23 ± 2 °C).

### 4.2. Preparation of 3D Constructs of Hybrid Hydrogels

The constructs developed in this work were ternary hydrogel scaffolds based on carboxymethylcellulose sodium salt (CMC, degree of substitution, DS = 0.84; average molecular mass, Mw = 700 kDa, Sigma-Aldrich, St. Louis, MI, USA), polyvinyl alcohol (PVA, Mw = 85–124 kDa, degree of hydrolysis, DH, 99.3–100%, Sigma-Aldrich, USA), and chitosan (CHI, Mw = 310–370 kDa e degree of deacetylation, DD, ~75%, Sigma-Aldrich, USA). The steps of 3D hydrogel preparation were described in the sequence.

Solutions of CMC and PVA (2% *w*/*v*) were prepared using magnetic stirring with heating at 60 ± 2 °C and 90 ± 5 °C, respectively, until complete solubilization of the polymer. A 2.0% *w*/*v* chitosan solution was prepared using a 2% *v*/*v* glacial acetic acid (Synth, São Paulo, SP, Brazil) solution with magnetic stirring at RT until the polymer was completely dissolved.

CMC (72 mL) and PVA (18 mL) solutions were mixed (4:1, *w*/*w* ratio) and homogenized under mechanical stirring for 30 min. Then, while maintaining stirring, the cross-linking agent citric acid was added at a concentration of 15% *w*/*w* (CA/(total polymer mass)) and homogenized for 1 h. After this period, 10 mL of the CHI solution was added, and the solution was maintained under vigorous mechanical stirring for 2 h. Immediately after, 10 mL of this solution was poured into plastic molds (60 mm diameter polystyrene Petri dishes) and dried at 40 ± 2 °C for 24 h to remove water. Subsequently, the samples were kept at 80 ± 2 °C for 24 h for the cross-linking reaction to occur. As a reference, a sample without CA was prepared and dried following the same thermal cycle. The produced samples were identified as CMC:PVA:CHI_CA and CMC:PVA:CHI (reference without citric acid, REF).

After the thermal treatment and cooling of the structures, they were removed from the molds and immersed in a phosphate-buffered saline solution (PBS, pH = 7.4. NaCl, KCl, Na_2_HPO_4_, and KH_2_PO_4_ reagents, Sigma-Aldrich, USA) for pH neutralization (range between 6.0 and 7.4). The process was repeated until the desired pH was reached.

After buffering, the samples were kept at −20 °C for 24 h, followed by a freeze-drying process for 24 h (Lyophilizer L108, LIOBRAS, São Carlos, SP, Brazil; equilibrium conditions 66 µHg and −53 °C). The freeze-dried samples were identified as CMC:PVA:CHI_FD.

### 4.3. Functionalization of 3D Constructs of Hybrid Hydrogels

A functionalization biomolecule (L-arginine, Sigma-Aldrich, USA) was grafted onto the hydrogel using 1-ethyl-3-[3-dimethylaminopropyl]carbodiimide (EDC, Sigma-Aldrich, USA) as a “zero-length” conjugation agent in a 2-(N-morpholino)ethanesulfonic acid (MES, Sigma-Aldrich, USA) buffer. The L-arginine (L-Arg) solution was prepared by adding amino acid powder (1.26 g) to 12 mL of 0.1 M HCl and adjusting the pH to approximately 5.5 using HCl solution (6 M, Sigma-Aldrich, USA). Then, 15 mL of MES buffer was added (0.2 M, pH 5.5 ± 0.1). The volume was finalized to 30 mL with DI water. The 3D structures were functionalized as follows: 10 mL of EDC solution was added to the Petri dish (polystyrene, 90 mm diameter) and the constructs were immersed in the EDC solution and incubated at RT for 15 min (1:2 molar ratio COO^−^:EDC) to activate the carboxyl groups of CMC.

Next, the EDC solution was removed, replaced with 10 mL of L-arginine solution (1:6 molar ratio COO^−^:L-arginine), and maintained for 20 h. Then, the functionalized hydrogels were washed with cold PBS solution (4–6 °C, pH = 7.4 ± 0.2) and immersed for 60 min in the same buffer. After, the samples were gently immersed in distilled water for 24 h and dried at 40 ± 2 °C for 24 h. The surface-functionalized hydrogels were identified as CMC:PVA:CHI_L-Arg.

### 4.4. Preparation of Tissue Engineered Skin Substitute Cellular 3D Scaffolds with Mono- and Co-Cultures of Cells

#### 4.4.1. Cells

The cell therapy products were produced using non-commercial allogeneic cells from human foreskin fibroblasts (h-FB cells, nh-skp FB0046) and human foreskin keratinocytes (h-KT cells, nh-skp—KT0088), where each cell was from one single donor, supplied by the Rio de Janeiro Cell Bank (BCRJ, Duque de Caxias, RJ, Brazil). The BCRJ is the biggest cell bank in South America, accredited by national and international standards and regulatory agencies for quality assurance (e.g., Organisation for Economic Co-operation and Development—OECD, European Centre for the Validation of Alternative Methods—ECVAM, and World Federation for Culture Collections—WFCC.; complying with ISO/IEC 17025:2017 [63], NIT DICLA 061:2018 [64], and ISO/TR 22758:2020 [65], Biotechnology/Biobanking/Implementation guide—ISO 20387:2018 [66]).

#### 4.4.2. Cutting/Sterilization/Pre-Treatment

The 3D structures were cut with a 6.4 mm diameter leather punch after swelling the materials for 15 min with PBS (1×) containing antibiotic and antifungal elements (penicillin G sodium, 10 units/mL; streptomycin sulfate, 10 mg/mL; amphotericin B, 0.025 mg/mL, PSA, Thermo Fisher, Waltham, MA, USA). After cutting and drying in the environment, the samples were sterilized by a UV-C source on both sides (low power, wavelength = 200–280 nm, distance ~50 mm, 2 h) [67,68]. Subsequently, the samples were placed in 96-well plates, and 100 µL of the specific medium for each cell type was added. For keratinocytes, Gold Keratinocyte Growth Basal Medium supplemented with a KGM-Gold^TM^ Keratinocyte Single Quots^TM^ Kit (Lonza, Morristown, NJ, USA), referred to as “complete basal keratinocyte medium”, was used. For fibroblasts, Dulbecco’s Modified Eagle Medium Low Glucose (DMEM, LGC Biotecnologia, Cotia, SP, Brazil) with 10% fetal bovine serum (FBS, Cripion Biotecnologia, Brazil) was used. In the sequence, samples were incubated for 24 h at 37 °C in an incubator in an atmosphere containing 5% CO_2_ before the cell seeding procedure for the 3D structures.

#### 4.4.3. Epidermal Skin Tissue Engineering Constructs: Keratinocyte Monoculture

Before seeding, keratinocytes were dissociated with the enzyme trypsin (Sigma-Aldrich, USA) to count and calculate the dilutions used in the experiment. Keratinocytes in complete basal keratinocyte medium were applied to the 3D structures on the upper surface of the sample (top side in the drying/cross-linking/lyophilization processes) at a concentration of 1 × 10^5^ cells/well and left in the incubator for 2 h to adhere to the samples. After this period, 100 µL of complete basal keratinocyte medium was added, followed by an incubation period of 3 days in an incubator at 37 °C and an atmosphere containing 5% CO_2_.

#### 4.4.4. Dermal Skin Tissue Engineering Constructs: Fibroblast Monoculture

Before seeding, fibroblasts were dissociated with trypsin to count and calculate the dilutions used in the experiment. Fibroblasts in DMEM medium with 10% FBS were applied to the 3D structures on the lower surface of the sample (bottom side, in contact with the Petri dish during drying/cross-linking/lyophilization) at a concentration of 9 × 10^5^ cells/well and left in the incubator for 2 h to adhere to the samples. After this period, 100 µL of DMEM medium with 10% FBS was added, followed by an incubation period of 3 days in an incubator at 37 °C and an atmosphere containing 5% CO_2_.

#### 4.4.5. Dermo-Epidermal Skin Tissue Engineering Constructs: Keratinocyte and Fibroblast Co-Culture

Before seeding, keratinocytes were dissociated with trypsin to count and calculate the dilutions used in the experiment. Keratinocytes in complete basal keratinocyte medium were applied to the 3D structures on the upper surface of the sample at a concentration of 1 × 10^5^ cells/well and left in the incubator for 2 h to adhere to the samples. After this period, 100 µL of complete basal keratinocyte medium was added, followed by a 24 h incubation period in an incubator at 37 °C and an atmosphere containing 5% CO_2_. After 24 h, the structures were flipped around, and fibroblasts were seeded in DMEM medium with 10% FBS at a concentration of 9 × 10^5^ cells/well. The plate was incubated for 3 days in an incubator at 37 °C and an atmosphere containing 5% CO_2_.

### 4.5. Characterization of Polymers and 3D Constructs of Hybrid Hydrogels

#### 4.5.1. Swelling Degree and Gel Fraction Tests

To measure the swelling degree (SD) and evaluate the gel fraction (GF), the hydrogels were cut into square samples (10 mm × 10 mm), dried at 40 ± 2 °C to stabilize mass, and then weighed (W_0_, initial mass). Subsequently, the hydrogels were placed in 10 mL of DI water at room temperature. DI water was chosen to avoid the chances of precipitation of buffer salts (e.g., PBS) in the highly porous 3D structure, which could affect the SD and GF measurements. After 24 h, the hydrogel was removed from the solution, gently wiped to remove excess liquid from the sample surface, and weighed (W_S_, swollen mass). Next, the samples were dried at 40 ± 2 °C until a constant mass was reached and weighed (W_f_, final mass), and the process was repeated for all samples. The SD and GF were determined according to Equations (1) and (2), respectively:

SD (%) = ((W_S_ − W_0_)/W_0_) × 100% (1)

GF (%) = ((W_0_ − W_f_)/W_0_) × 100% (2)

#### 4.5.2. Spectroscopic and Morphological Analyses

Nuclear Magnetic Resonance (NMR) spectra of polymers were obtained using an Avance III-400 MHz spectrometer (Bruker, Billerica, MA, USA; 90 pulses, 16 scans) at 30 °C. All samples were prepared in a D_2_O medium, but, to obtain the CHI solution, HCl was also added, aiming to provide an acidic condition to improve sample solubilization. ^1^H NMR spectra were collected for CMC, CHI, and PVA, whereas ^13^C NMR was also performed for PVA. Water signals were suppressed in all analyses.

Fourier-transform infrared spectroscopy (FTIR) analyses were performed using a Nicolet 6700 spectrometer (Thermo Fisher, USA) with background noise subtraction. The FTIR spectra of the polymers and hydrogels were collected based on the attenuated total reflectance method (ATR) within the wavenumber range of 4000–750 cm⁻^1^, using 32 scans and a resolution of 4 cm⁻^1^.

X-ray photoelectron spectroscopy analysis (XPS, Amicus, Kratos, Japan) was performed using a Mg-Kα X-ray source at 120 W. The spectra were calibrated using the C-C/C-H peak from the high-resolution C 1s spectrum of silver set to 284.5 eV.

Scanning electron microscopy images (SEM) were collected from the surfaces of the hydrogels using an FEI Inspect S50 microscope (FEI Company, Hillsboro, OR, USA). Before the SEM analysis, as the hydrogels were not conductive, all samples were coated with an ultra-thin gold-sputtered film, deposited at a low rate to prevent sample damage and charge-up under the electron beam.

The three-dimensional structures of the scaffolds were also investigated using X-ray microtomography (SkyScan 1174, Bruker, Coventry, UK) at a resolution of 12.18 μm, with a voltage of 35 kV, current of 800 μA, and rotation step of 0.7°, without filters. The digital images were reconstructed using NRecon Reconstruction software (v.1.6.1.18, Bruker micro-CT). CTAn software (v.1.15.4.0, Bruker micro-CT) was used to analyze micro-CT datasets in 2D and 3D for morphometry and densitometry, while CTVox was used for the 3D visualization of the scaffolds.

#### 4.5.3. Measurements of Thickness, Wettability, and Surface Charge

The thickness of the 3D structures was measured using a caliper (Mitutoyo, Kawasaki, Japan). The results were based on five measurements and are expressed as mean ± standard deviation. The wettability of the hydrogels was assessed by measuring the contact angle (ϕ) formed between DI water and the hydrogels. To obtain the contact angle measurements, a drop of deionized water was placed on the surface of each hydrogel using a microsyringe at room temperature, and digital images were acquired immediately after deposition (<5 s). The results represent the average angle between the tangent line at the droplets and the surface of the hydrogel membranes.

The evaluation of the point of zero charge (PZC) was conducted by immersing membranes/scaffolds weighing approximately 8.0 ± 0.5 mg in vials containing 10 mL of NaCl solution (0.01 M, Sigma-Aldrich, USA), with the pH adjusted in the range of 4 to 9 using 1.0 M and 0.1 M NaOH and HCl solutions (Sigma-Aldrich, USA). The vials with the membranes were maintained under gentle stirring (100 rpm) for 20 h, and the final pH values of each vial were assessed. The difference between pH values, i.e., final and initial pH (=ΔpH), for each system was plotted against the initial pH, and the PZC value was considered the point where ΔpH = 0.

#### 4.5.4. Cytotoxicity Tests: In Vitro Bioassays

Cytotoxicity tests were performed using human foreskin fibroblasts (h-FB cells, nh-skp FB0046) and human foreskin keratinocytes (h-KT cells—nh-skp—KT0088) provided by the Rio de Janeiro Cell Bank (BCRJ, Brazil).

The cytocompatibility bioassays of the hydrogels were conducted according to the most accepted international standard (ISO 10993-5:2009) for the biological evaluation of medical devices. Prior to the experiments, the samples were sterilized by exposure to conventional UV-C radiation, as previously described.

The cytotoxicity of the hydrogels was evaluated by the direct contact method with 3-(4,5-dimethyl-2-thiazolyl)-2,5-diphenyl-2H-tetrazolium bromide (MTT, Sigma-Aldrich, USA). The h-FB cells were cultured and tested in Dulbecco’s Modified Eagle Medium (pH 7.4 ± 0.2, LCG Biotechnology, Brazil) with the addition of 10% fetal bovine serum (FBS, Cripion Biotechnology, Andradina, SP, Brazil) and antibiotic and antifungal elements (PSA, Thermo Fisher, USA). The h-KT cells were cultured and tested in a complete basal keratinocyte medium (Lonza, USA). Both cell lines (h-FB and h-KT) were cultured at T = 37 °C in a 5% CO_2_ atmosphere.

The h-FB (passage 4) and h-KT (passage 4) cell lines were plated (3 × 10^5^ cells/well) in 96-well plates and the cell population was synchronized for 24 h. Then, the cells were incubated in contact with hydrogel samples (1 mg/well) for 24 h at 37 °C. Then, the total volume in each well was aspirated, and it was replaced with 100 µL of culture media with MTT reagent (5 mg/mL). These samples were incubated for 4 h (T = 37 °C, atmosphere of 5% CO_2_).

Next, 40 µL sodium dodecyl sulfate (SDS, Sigma Aldrich, USA) with 4% HCl (Sigma Aldrich, USA) was poured into each well. Then, samples were incubated in an oven for 16 h (37 ^°^C, atmosphere of 5% CO_2_). In the sequence, from each well, a volume of 100 µL was precisely transferred to a similar blank 96-well plate for absorbance measurements.

Control samples were designed as follows: the control was cell culture with a specific medium; the positive control (+ Control) was cell culture with a specific medium and 1.0% *v*/*v* Triton™ X-100 (Sigma-Aldrich, USA); and the negative control (−Control) was cell culture with specific medium and sterile polypropylene chips at 1 mg/well (Eppendorf^®^, Hamburg, Germany).

The percentage of cell viability was estimated according to Equation (3) using the absorbance (Abs) values of the control and samples measured at 595 nm (Varioskan™ LUX multimode microplate reader, Thermo Scientific, Waltham, MA, USA). The values of the control were considered as 100% of the cell viability response.

Cell viability (%) = (Abs_(sample)_/Absorbance_(control)_) × 100% (3)

#### 4.5.5. Cell Proliferation

In the resazurin assay, fibroblasts (passage 11) and keratinocytes (passage 4) were seeded on 3D scaffolds (Section 4.4.3 and Section 4.4.4) and, after 3 days of incubation, the medium was replaced by 180 μL of a specific culture medium. Then, 20 μL of resazurin stock solution (0.1 mg/mL, Sigma-Aldrich, USA) was added to each well and incubated for 2 h (T = 37 °C, *p* = 5% CO_2_ atmosphere). In the sequence, 100 μL was removed from each well and transferred to a 96-well plane, and the resorufin fluorescent intensity was measured using the Varioskan™ LUX multimode microplate reader (Thermo Scientific, USA; λ_excitation_ = 570 nm, and λ_emission_ = 590 nm). PL intensity values were expressed as a fluorescence intensity (%) relative to the control wells (100%). Data were presented as the mean and standard deviation of five replicates (n = 5).

#### 4.5.6. LIVE/DEAD Cell Staining

To assess the cell viability and adhesion, cells were stained using a LIVE/DEAD Viability/Cytotoxicity Kit (Thermo Fisher Scientific, USA). Fibroblasts (passage 11) and keratinocytes (passage 4) were seeded on 3D hydrogels according to the methods described in Section 4.4. After the cells’ cultivation period, the 3D systems were stained with calcein AM for live cells, and dead cells were marked with ethidium homodimer-1, according to the manufacturer’s protocol. The digital images of stained cells were captured by a Ti-U epifluorescence microscope (Nikon Instruments, New York, NY, USA) using a FITC filter cube (λ_excitation_ = 480/30 nm and λ_emission_ = 515 nm) for live cell green emission. Images of dead cells (red emission) were acquired using a Texas Red filter cube (λ_excitation_ = 560/40 nm and λ_emission_ = 635/60 nm).

#### 4.5.7. Hemocompatibility

The hemolytic index (HI) was applied to evaluate the hemocompatibility of the samples. Hydrogels with dimensions of 1.0 cm × 1.0 cm were immersed for 1 h in PBS solution at 37 °C (Sigma-Aldrich, USA) for medium absorption and swelling. Subsequently, the PBS solution was removed, and 250 μL of mouse blood (BALB/c isogenic adult male mice, protocol approved by the Ethics Committee CEUA-UFMG, no. 22/2018) with heparin (4 IU/mL; Cristália, Belo Horizonte, MG, Brazil) was added. The systems were left undisturbed for 20 min and then the hemolysis process was halted by adding 1.75 mL of 0.9% NaCl saline solution (Sigma-Aldrich, USA). These samples were incubated (37 °C/1 h). Then, samples and controls were centrifuged (4000 rpm/10 min) and the absorbance (Abs) of the supernatant was measured at λ = 545 nm (Spectramax M, Molecular Devices, San Jose, CA, USA). Hemolytic index values were calculated according to Equation (4).

HI (%) = [(Abs_(sample)_ − Abs_(−Control)_/(Abs_(+Control)_ − Abs_(−Control)_] × 100% (4)

where “−Control (negative control)” is a mixture of 250 μL of blood and 1750 μL of 0.9% NaCl saline solution and “+Control (positive control)” is a mixture of 250 μL of blood and 1750 μL of bidistilled water.

#### 4.5.8. Statistical Analysis

Prism software (Version 8.0.2, GraphPad Software, La Jolla, CA, USA) was used for data analysis. Statistical analysis was performed considering a *p*-value of less than 0.05 as significant, and the results are expressed as mean ± standard deviation. The experiments were conducted with n ≥ 3, and the method used was one-way ANOVA with the Bonferroni method.

### 4.6. In Vivo Assays for Wound Healing in an Animal Model

#### 4.6.1. Experimental Design

In this study, all in vivo experiments were performed according to the project approved by the Ethics Committee on Animal Research from the Universidade Federal de Minas Gerais (CEUA, UFMG; protocol certificate #312/2024).

In this study, 6- to 7-week-old female Hairless (hr/js) mice were used. The mice were accordingly housed and monitored in the animal facility of the Laboratory of Immunobiology and Control of Parasites, Department of Parasitology-UFMG, and kept under 12 h/12 h dark–light cycles. All mice had free access to dry food and water ad libitum.

Hairless (Nude) animals were used as an experimental model of immunodeficiency due to their deficient T-lymphocyte response caused by the complete absence (or atrophy) of the thymus, allowing for the testing of cell therapy products seeded with human cells [69].

#### 4.6.2. Induction of Tissue Injury and Application of Therapeutic Products

The experimental surgical procedure for the allocation of the membranes was performed after trichotomy of the dorsal part of the animals. Once shaved, the animals were anesthetized intraperitoneally using 2% xylazine (Syntec, Barueri, SP, Brazil) and 5% ketamine (Syntec, Brazil). After establishing anesthesia, the animals were placed in the prone position, and the midline in the dorsal region was cleaned with a swab soaked in 70% ethanol (NEON, São Paulo, SP, Brazil).

The skin wound was produced in the previously delimited region with the aid of a biopsy punch and sterile scalpel to form a circular lesion with an approximate diameter of 5 mm and depth of 1–2 mm, depending on the thickness of the skin of the mice specimens. The depth of the wound included the epidermis, dermis, and hypodermis, going through the surface of the muscular fasciae (full-thickness open wound). In cases where minor bleeding occurred, this was controlled through local compression with the aid of sterile gauze. The size of each lesion was measured (length × width) with an automated caliper. After the allocation of the sample membrane, the area was protected with circular blood-stop dressings with a diameter of 25 mm. The cellular skin substitute was applied to the wound bed only one time and remained there for 14 days during the in vivo experiment.

#### 4.6.3. Assessment of the Efficiency of Therapeutic Products in Healing Injuries

After injury induction and membrane application, the animals were monitored for up to 14 days. During this period, the body weight was continuously monitored, and the wounds of all animals were photographed (distance of the camera from animal ~5 cm) and clinically evaluated, with the presence of inflammatory signs (edema and hyperemia) registered when applicable. In addition, the diameter of the lesions was estimated with a digital caliper (length: craniocaudal measurement and width: side-to-side measurement). Based on Equation (5), the percentage of wound healing (WHR, %) was calculated, as previously described [70]:

Wound Healing (%) = 1 − (A_x_/A_1_) × 100 (5)

where A_1_ is the initial area (mm^2^) determined on day zero (D0—day of injury induction) and A_x_ represents the measurement of the wound area (mm^2^) obtained in the subsequent days of evaluation (D_x_).

#### 4.6.4. Euthanasia and Tissue Collection

Animals were euthanized by prior application of anesthesia intraperitoneally with a ketamine (100 mg/kg) and xylazine (10 mg/kg) solution. Afterward, with the aid of sterile surgical materials, tissue was collected from the entire region of the wound (including the edge of the wound) for subsequent histopathological analysis.

For histopathological analysis, samples from the lesion areas were fixed in 10% formaldehyde (NEON, Brazil) and gradually dehydrated in ethanol. Subsequently, they were cleared in xylene and included in paraffin blocks, being subsequently sectioned in 4–5 µm thick sections, followed by fixation on the microscopy slide.

The slides produced were stained with hematoxylin and eosin (NEON, Brazil) and qualitatively evaluated to verify tissue regeneration profiles (considering scar progression, fibroblastic proliferation, inflammatory foci, and neovascularization).

#### 4.6.5. Statistical Analysis

The results of in vivo assays are expressed as mean ± standard deviation. A two-way ANOVA variance test was performed to analyze the differences between the groups. Statistically, *p*-values lower than 0.05 were considered significant.

## Data Availability

All relevant data are available in the manuscript.
